# Predictors of right ventricular function as measured by tricuspid annular plane systolic excursion in heart failure

**DOI:** 10.1186/1476-7120-7-51

**Published:** 2009-11-04

**Authors:** Jesper Kjaergaard, Kasper K Iversen, Dilek Akkan, Jacob Eifer Møller, Lars V Køber, Christian Torp-Pedersen, Christian Hassager

**Affiliations:** 1 The Heart Centre, Copenhagen University Hospital, Rigshospitalet, Copenhagen, Denmark; 2 Department of Cardiology, Copenhagen University Hospital, Gentofte Hospital, Hellerup, Denmark

## Abstract

**Introduction:**

Tricuspid Annular Plane Systolic Excursion (TAPSE) has independent prognostic value in heart failure patients but may be influenced by left ventricular (LV) ejection fraction. The present study assessed the association of TAPSE and clinical factors, global and regional LV function in 634 patients admitted for symptomatic heart failure.

**Methods & Results:**

TAPSE were correlated with global and regional measures of longitudinal LV function, segmental wall motion scores and measures of diastolic LV function as measured from transthoracic echocardiography.

LV ejection fraction, wall motion index scores, atrio-ventricular annular plane systolic excursion of the mitral annulus were significantly related to TAPSE. Septal and posterior mitral annular plane systolic excursion (β = 0.56, p < 0.0001 and β = 0.35, p = 0.0002 per mm, respectively) and non-ischemic etiology of heart failure (β = 1.3, p = 0.002) were independent predictors of TAPSE, R^2 ^= 0.28, p < 0.0001. The prognostic importance of TAPSE was not dependent of heart failure etiology or any of the other clinical factors analyzed, p_interaction _= NS.

**Conclusion:**

TAPSE is reduced with left ventricular dysfunction in heart failure patients, in particular with reduced septal longitudinal motion. TAPSE is decreased in patients with heart failure of ischemic etiology. However, the absolute reduction in TAPSE is small and seems to be of minor importance in the clinical utilization of TAPSE whether applied as a measure of right ventricular systolic function or as a prognostic factor.

## Introduction

Right ventricular (RV) dysfunction is an important entity in heart failure as patients with reduced RV ejection fraction have poorer exercise tolerance and prognosis than patients with preserved RV function regardless of left ventricular (LV) function and degree of pulmonary hypertension [1]. Tricuspid annular plane systolic excursion (TAPSE) has been proposed as a simple and reproducible parameter for quantitative assessment of RV ejection fraction [2]. The prognostic importance of TAPSE in the evaluation of RV function in patients with severe heart failure has been well-described [3], and the parementer has been recommended in the most recent joint American European guidelines for echocardiographic quantification of right ventricular function [4]. Later studies showed the prognostic information in a mixed heart failure patients as well [5]

More recent validation studies have confirmed the relation between RV ejection fraction and TAPSE in various heart failure populations, although the association is modest in patients with preserved RV function [6-8]. In addition, it has been suggested that TAPSE may not be entirely independent of LV function [9].

The present study investigated the relation of TAPSE and other echocardiographic and clinical parameters, with emphasis on measures of regional LV function and clinical characteristics of heart failure patients.

## Methods

The present study is a sub-study based on the population of Danish patients, consecutively screened for participation in the EchoCardiography and Heart Outcome Study (ECHOS). The ECHOS trial was a prospective, double-blind randomized, placebo-controlled multi-centre trial of a selective agonist of the pre-synaptic DA_2 _and α_2 _receptors. The trial was based on a screening population defined as consecutive patients, ≥ 18 years of age, admitted for symptomatic heart failure corresponding to New York Heart Association (NYHA) class III-IV within the last month, requirering treatment with diuretics. Patients were not eligible for screening if an acute myocardial infarction was diagnosed. Screening for the ECHOS trial included a transthoracic echocardiography (TTE) performed at rest in the left lateral position according to a predefined protocol ensuring standardized echocardiographic imaging for the assessment of LV ejection fraction by wall motion index (WMI) scoring. In addition to the echocardiographic imaging, demographic and clinical data were collected. All patients screened for participation in the trial was included in the present analysis since no effect on the primary end-point (overall mortality) in the randomized study was observed [10]. The ECHOS study complied with the principles in the Declaration of Helsinki II, and was approved by the regional ethics committee prior to initiation. All patients provided written informed consent before screening for the ECHOS trial was preformed.

All centers were encouraged to record additional predefined echocardiographic views and standardized training of physicians from the participating centers was performed prior to initiation of screening. Imaging were stored on videotapes and digitized at a core laboratory (MPEGator, Darim Vision Corp., Pleasanton, CA, USA). All measurements were performed using electronic calipers on custom-made software following on-screen calibration, and were reported as the average of five measurements performed by two experienced readers, D.A. and J.K. The echocardiographic imaging included three apical views for assessment of LV function by WMI (hyperkinesis = 3, normokinesis = 2, hypokinesis = 1, akinesis = 0, paradoxical motion = -1) in a 16 segment model [11], which allowed approximation of the LV ejection fraction from average WMI by multiplication of 30 [12]. Optional imaging included views for measurements right ventricular end-diastolic outflow tract dimensions in the parasternal long axis view and tricuspid regurgitation velocity measured by continuous wave Doppler in the apical 4-chamber view, which was used for calculation of tricuspid regurgitation pressure gradient by the modified Bernoulli equation [13]. TAPSE was measured by M-mode echocardiography at the junction of the tricuspid valve and RV free wall in the apical 4 chamber view as previously illustrated [5]. LV parameters included views for measurements of LV diameters, systolic function by mitral valve atrio-ventricular plane systolic excursion at the septal, lateral, anterior and posterior annulus [14], diastolic function by mitral valve inflow profile for early (E) and atrial (A) velocities, the E/A ratio, early inflow deceleration time and atrial size by M-mode in the parasternal long axis view. The coefficient of variation between independent measurements of TAPSE was 10% (duplicate measurements, n = 20).

Baseline data recorded at screening included known cardiovascular risk factors associated with HF: demographic data, current tobacco use, diabetes, paroxysmal atrial fibrillation, and co-existing cardiovascular disease including arterial hypertension, ischemic heart disease. The local investigator also recorded the presence of Chronic Obstructive Pulmonary Disease (COPD) and chronic reduced renal function. Presumed heart failure etiology was reported by the local investigator as ischemic or non-ischemic (hypertensive, valvular, hypertrophic, idiopathic cardiomyopathy or other/unknown).

We have previously shown that TAPSE is an independent predictor of all-cause mortality in the present population [5]. We sought to determine whether clinical factors or co-morbidities potentially influencing TAPSE would have an impact on the prognostic value of TAPSE.

### Statistical analysis

Data was presented as mean ± standard deviation (SD) or number (%). The relations of parameters to TAPSE were tested by linear regression analysis for continuous variables and by *t*-test for categorical variables. Normal distribution of the continuous values was assessed by the Kolmogorov-Smirnov test. Multivariate linear regression models including all significant clinical and echocardiographic parameters from the univariate analysis were constructed to assess independent predictors of TAPSE. Survival analysis for the assessment of impact of differences in the etiology of heart failure on TAPSE was performed by proportional hazard regression analysis as previously described testing for the interaction of TAPSE and co-variates [5]. SAS software (Cary, NC) version 9.1 was used for all computations, and a p-value < 0.05 was considered statistically significant.

## Results

**Figure 1:**
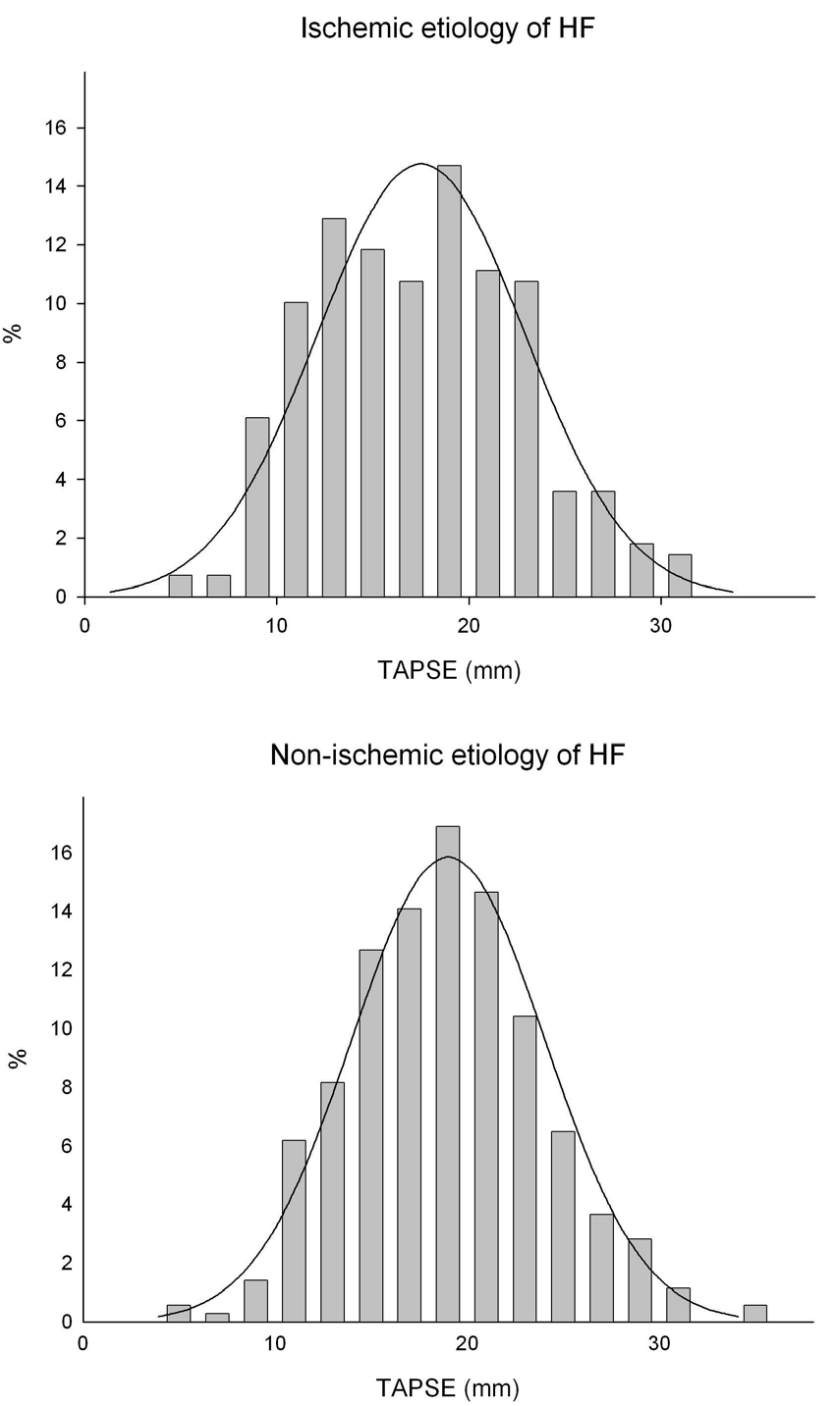
**Histogram of the relative prevalence of measures of TAPSE in 634 patients admitted for symptomatic heart failure, stratified by presumed etiology of heart failure: Ischemic (N = 279) or Non-ischemic (N = 355)**. Lines represent the normal distribution of the mean in the sample.

None of the demographic and anthropomorphic parameters available were related to values of TAPSE, whereas several of the clinical parameters were significantly related to TAPSE, Table 1. These factors included heart failure etiology where patients with Ischemic cardiomyopathy were found to have lower values of TAPSE compared to non-ischemic etiology (17 ± 5 mm vs.19 ± 5 mm, p = 0.001), see Table 1. As illustrated in Figure 1 the distribution of values of TAPSE was normal in HF patients with non-ischemic etiology, whereas the distribution in patients with ischemic etiology of heart failure had a less homogeneous appearance, although TAPSE were normally distributed in both groups, p_Kolmogorov-Smirnov _= 0.08 and p_Kolmogorov-Smirnov_>0.15, respectively.

**Table 1:** Clinical demographic characteristics and medical history in the study population and relation to TAPSE in univariable linear regression analysis

	**Mean ± SD or** **N (%)**	**Mean ± SD**	**β**	**p-value**
Age (per years)	72 ± 12		0.01	0.53
Sex, male, n (%)	388 (61)	18.8 ± 4.7	-0.8	0.05
Height (cm)	170 ± 9		0.01	0.69
Weight (kg)	76 ± 18		0.02	0.11
BMI (kg/m^2^)	26 ± 5		0.06	0.20
Heart rate	92 ± 26		-0.02	0.004
Medical history				
Ischemic etiology of heart failure	279 (44%)	19.2 ± 5.0	-1.7	0.0003
Hypertension	167 (26%)	18.3 ± 5.2	0.4	0.43
Heart Failure, previously diagnosed	423 (67%)	19.2 ± 4.9	-1.3	0.002
Paroxysmal atrial fibrillation	157 (25%)	18.7 ± 5.4	-1.4	0.005
Diabetes, type I or II	95 (15%)	18.4 ± 5.2	-0.1	0.83
Chronic Obstructive Pulmonary disease	140 (22%)	18.2 ± 5.2	1.0	0.05
Smoking, current	193 (31%)	18.0 ± 5.1	1.1	0.01
Reduced renal function	67 (11%)	18.4 ± 5.2	-0.3	0.66

**Figure 2:**
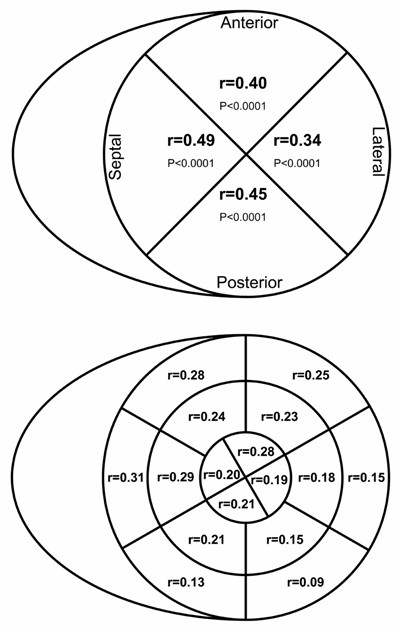
**Bulls-eye plot of the correlation of Tricuspid Annular Plane Systolic Excursion (TAPSE) and measures of left ventricular systolic performance: Mitral atrio-ventricular plane systolic excursion at the septum, lateral, anterior and posterior wall (top panel) and 16 segment wall motion analysis (lower panel), p < 0.05 for all**.

Echocardiographic predictors of TAPSE were global LVEF, deceleration time of the early mitral inflow (E), the late mitral inflow (A) and E/A in univariate analysis, see Table 2. Detailed analysis of the regional longitudinal motion of the mitral annulus in relation to TAPSE revealed a highly significant direct correlation, see Table 2 and Figure 2. The association seemed stronger in the septal compared to the lateral and in posterior compared to the anterior left ventricular wall, see Figure 2 (top panel). Regional radial motion, as quantified by wall motion index score, was also significantly correlated to TAPSE, and the relation seemed stronger in the basal segments compared to the apical segments and in the septal and anterior-septal segments than in the lateral or inferior segments, see Figure 2 (bottom panel). Overall the longitudinal LV motion was stronger related to TAPSE than was radial motion. No correlation of measures of left or right chamber size or systolic pulmonary pressure were found, see Table 2.

**Table 2:** Univariable analysis of the relation of Echocardiographic parameters and TAPSE

	**Mean ± SD**	**β**	**p-value**
Left ventricular parameters			
Left ventricular end-diastolic diameter (mm)	59 ± 10	-0.04	0.18
Ejection fraction by WMI (%)	38 ± 16	0.09	<0.0001
LV atrio-ventricular plane systolic excursion, Septal (mm)	7.4 ± 3.1	0.83	<0.0001
LV atrio-ventricular plane systolic excursion, Anterior (mm)	8.4 ± 3.3	0.63	<0.0001
LV atrio-ventricular plane systolic excursion, Posterior (mm)	8.6 ± 3.2	0.7	<0.0001
LV atrio-ventricular plane systolic excursion, Lateral (mm)	10.1 ± 3.6	0.51	<0.0001
Left atrial diameter (mm)	39 ± 6	-0.09	0.06
E deceleration time (msec)	157 ± 68	0.02	0.003
E peak velocity (m/s)	0.92 ± 0.29	-0.68	0.41
A peak velocity (m/s)	0.73 ± 0.31	4.43	<0.0001
E/A ratio	1.4 ± 0.8	-1.3	0.0002
Right ventricular parameters			
Tricuspid regurgitation pressure gradient (mmHg)	33 ± 14	0.003	0.93
Right ventricular diameter (mm)	32 ± 6	-0.07	0.22

Multivariable analysis showed that septal and posterior longitudinal motion and HF etiology were independently related to TAPSE, see Table 3. LVEF was different in the two groups of etiology of heart failure (35 ± 14 and 41 ± 16% for ischemic and non-ischemic etiology, respectively, p < 0.0001), but there were no effect of this difference on the relation of HF etiology and TAPSE, p_interaction _= not significant (NS).

**Table 3:** Multivariable analysis of clinical and echocardiographic predictors of TAPSE in heart failure patients.

	**β (95% CI)**	**p-value**
LV atrio-ventricular plane systolic excursion, Septal (mm)	0.56 (0.36-0.76)	<0.0001
LV atrio-ventricular plane systolic excursion, posterior (mm)	0.35 (0.17-0.54)	0.0002
Heart failure etiology		
Non-ischemic vs. ischemic etiology of heart failure	1.3 (0.5-2.2)	0.002

The original model included all significant parameterS in Tables 1 and 2. R^2 ^of the final model = 0.28.

While the independent prognostic value of TAPSE in the present population has previously been shown [5], the factors identified to be associated with TAPSE in the present study could be potential confounding factors with regards to the association of TAPSE and mortality. We therefore repeated the survival analysis including all of these factors and found no impact of heart failure etiology on the prognostic significance of TAPSE or any of the other clinical factors listed in Table 1, p_interaction _= NS.

## Discussion

TAPSE is a widely recognized, clinically useful and feasible marker of right ventricular dysfunction, and has been proven to be a valuable prognostic marker in various cardiac diseases, including heart failure. The present study adds that TAPSE may be influenced by left ventricular septal longitudinal motion in patients with symptomatic heart failure patients and that TAPSE is affected by the etiology of heart failure. None of identified factors however, induces significant limitation in the every day use of TAPSE as a prognostic marker in the assessment of heart failure patients.

Reduced LVEF seem to have an impact on TAPSE, even in the setting of preserved right ventricular ejection fraction [9,15]. The present study adds that longitudinal motion of the septal and adjacent segments are closer related to TAPSE than radial motion as assessed by wall motion and lateral segments, respectively. Furthermore, basal segments seem stronger correlated to TAPSE than apical segments. The concept of ventricular interdependence shown in experimental models could thus be an important explanatory factor in the relation of TAPSE and LVEF [16]. TAPSE was also found to be correlated to markers of diastolic dysfunction although none of these parameters were independent parameters in the multivariable model. RV end-diastolic diameter and systolic pressure as estimated by tricuspid regurgitation pressure gradient were not related to TAPSE as previously reported [5], while an earlier study showed a weak correlation of pulmonary artery pressure and RV function in heart failure patients [1]. The mean tricuspid regurgitation pressure gradient was 33 mmHg in the present study, and it could be considered low in a symptomatic heart failure population, but should be interpreted in the context of being measured after treatment with diuretics have been initiated.

Pulmonary disease and heart failure share a number of risk factors and as a result, co-existing pulmonary disease in heart failure patients is reported in about 25% of patients [17]. In addition heart failure per se is associated with both restrictive and obstructive changes in pulmonary function [18,19]. Presence of COPD in the medical history was not related to TAPSE in the present study, and earlier studies have shown that RV dysfunction only occur at advances stages of the disease [20,21].

Several clinical factors were found to be related to decreased TAPSE although the reduction on average was in the order of 1 mm, and thus likely to be of limited clinical importance. These factors included a history of ischemic heart disease, previously diagnosed heart failure and paroxysmal atrial fibrillation and presumed ischemic origin of heart failure, of which only the latter was found to be an independent predictor of TAPSE when adjustments for echocardiographic parameters were made. TAPSE is known to be reduced in patients with atrial fibrillation and to normalize after conversion to sinus rhythm [22], and the present study adds that presence of paroxysmal atrial fibrillation is also related to reduced TAPSE. Previous studies have reported that non-ischemic cardiomyopathy more frequently affects both ventricles than heart failure of ischemic origin [23,24]. In one study TAPSE was actually increased in patients with anterior myocardial infarction as a compensatory mechanism [25]. In contrast, TAPSE is reduced in patients with infarct involving the RV compared to patients with anterior or inferior myocardial infarction without electrocardiographic evidence of RV involvement [26]. In accordance we found a heterogeneous distribution of TAPSE in patients with ischemic etiology, which could suggest a different impact on TAPSE depending on location or extent of previous infarcts. Unfortunately, data on coronary anatomy was not available in the present study, but could be assessed in future studies to further clarify this subject. No influence of etiology of HF on the prognostic importance of TAPSE was found, which further consolidates the use of TAPSE in echocardiographic examination of HF patients.

TAPSE has previously been found to be independent of age and gender in healthy individuals [27,28], and the present study adds that TAPSE is also unrelated to age and gender patients with heart failure. The lack of relation to body size also consolidates the routine use of TAPSE without correction for body surface area in these patients. Tachycardia on the other hands seems to be associated with reduced values of TAPSE, and interpretation of TAPSE in presence of tachycardia should be performed with caution.

### Limitations

Heart failure etiology and presence of co-morbidity was reported by the local investigator, and although pre-specified definitions for these factors were provided, the accuracy of these data may be less than perfect. However, we find it unlikely that this would induce significant bias in the present analysis.

Imaging for the centralized echocardiographic analysis was performed at the local centers. To limit variation, physicians from all centers received training in the acquisition of all pre-specified views included the optional imaging of the measures reported in the presents study. Differences between patients in whom imaging for analysis of TAPSE was not available and the present cohort have been previously described [5], and we find it unlikely to be a significant source of confounding in the present analysis.

## Conclusion

TAPSE is reduced with left ventricular dysfunction in heart failure patients, in particular with reduced septal longitudinal motion. TAPSE is decreased in patients with heart failure of ischemic etiology. However, the absolute reduction in TAPSE is small and seems to be of minor importance in the clinical utilization of TAPSE whether applied as a measure of right ventricular systolic function or as a prognostic factor.

## Competing interests

The authors declare that they have no competing interests.

## Authors' contributions

JK and DA performed the analysis of the echocardiographic material. JK drafted the manuscript and performed initial statistical computations. KKI, JEM, LVK, CTP and CH had contributions to the statistical analysis and presentation of data. All authors read and approved the final manuscript.
